# Influence of the Perspectives on the Movement of One-Leg Lifting in an Interactive-Visual Virtual Environment: A Pilot Study

**DOI:** 10.1371/journal.pone.0163247

**Published:** 2016-09-20

**Authors:** Chien-Hua Huang, Chun Pei, Tien-Lung Sun

**Affiliations:** 1 Department of Industrial Engineering & Management, Yuan Ze University, Chung-Li, Taiwan; 2 Feng Yuan Hospital, Ministry of Health and Welfare, Taichung City, Taiwan; 3 Department of Geronic Technology and Service Management, Nan Kai University of Technology, Nantou County, Taiwan; Duke University, UNITED STATES

## Abstract

**Background:**

Numerous studies have confirmed the feasibility of active video games for clinical rehabilitation. To maximize training effectiveness, a personal program is necessary; however, little evidence is available to guide individualized game design for rehabilitation. This study assessed the perspectives and kinematic and temporal parameters of a participant’s postural control in an interactive-visual virtual environment.

**Methods:**

Twenty-four healthy participants performed one-leg standing by leg lifting when a posture frame appeared either in a first- or third-person perspective of a virtual environment. A foot force plate was used to detect the displacement of the center of pressure. A three-way mixed factor design was applied, where the perspective was the between-participant factor, and the leg-lifting times (0.7 and 2.7 seconds) and leg-lifting angles (30°and 90°) were the within-participant factors. The reaction time, accuracy of the movement, and ability to shift weight were the dependent variables.

**Results:**

Regarding the reaction time and accuracy of the movement, there were no significant main effects of the perspective, leg-lifting time, or angle. For the ability to shift weight, however, both the perspective and time exerted significant main effects, F(1,22) = 6.429 and F(1,22) = 13.978, respectively.

**Conclusions:**

Participants could shift their weight more effectively in the third-person perspective of the virtual environment. The results can serve as a reference for future designs of interactive-visual virtual environment as applied to rehabilitation.

## Introduction

Standing on a single leg by lifting the opposite leg is often used in clinical tests of balance, including the one-leg-standing (OLS) test [1], the Berg Balance Scale [2], and choice stepping reaction time [3]. Shorter OLS times or stepping reaction times indicate an increased risk of falling [1, 3]. Therefore, one-leg lifting training is crucial for balance.

Stepping exercises are common in traditional rehabilitation training because this type of functional practice is considered beneficial for ambulation. For example, a therapist might teach patients to step over obstacles that they might encounter when walking outside. A sidewalk curb is approximately 20 cm high; however, because the height may vary, patients should practice lifting their leg higher than this curb height. In addition to height, patients must walk within a fixed time, as when crossing a street at a traffic light. The optimal training model must entail real-life contingencies, which are difficult to recreate. Combining virtual reality (VR) and motion capture technology can solve this problem by providing additional sensory feedback and different custom-made environments. Numerous studies have confirmed the feasibility and effectiveness of active video games, such as the functional recovery of upper and lower extremities for post-stroke patients [4, 5]. Although many therapists understood the advantages of active video games, they reported difficulty finding time to learn how to use them [6, 7]. Thus, a simple, quick platform must be developed.

Regarding game design, two considerations are paramount: perspective and parameters. The two perspectives used in games are first-person (1PP) and third-person (3PP). In the 1PP perspective, the player sees through the eyes of the character or object that he or she controls, whereas in the 3PP, the player sees an avatar onscreen [8]. Prasad and Shiffrar [9] reported that people can identify their own movement more easily in 3PP than in 1PP, despite having little real-world experience seeing themselves in 3PP. In rehabilitation, therapists typically place a mirror in front of patients to facilitate learning movements. The patients can observe their movements concurrently and determine how they can move more easily [10]. Therefore, 3PP may be more helpful for rehabilitation training. Additionally, certain parameters must be set. Koritnik et al. [11] conducted a preliminary investigation to assess the feasibility of a virtual mirror system for lower-extremity training. In that study, a healthy participant saw his or her own figure superimposed on that of a virtual instructor, and attempted to match the instructor’s cadences and knee-joint lifting heights. Ustinova et al. [12] created an avatar in a virtual environment (VE) to analyze body movement and performance at different arm-reaching angles. Kinematics data were collected, and the results showed that reach distance was greatest at angles between 45°and 77.5°, without a corresponding loss of balance. Thus, manipulating kinematic or temporal parameters in VR may provide useful information for game design or rehabilitation programming.

### Theoretical framework

Patients recovering from stroke often experience difficulty when applying weight to their hemiplegic leg. Traditionally, to facilitate shifting weight, patients are first asked to place their sound foot on a small step in front of them. The height of the step is increased, and patients are asked to lift their sound foot higher as their balance improves [13]. As mentioned, patients are trained to step over a sidewalk curb. Based on the information processing model [14], they look at the curb first (sensory input), determine how distant and how high the curb is (perceptual stage), decide whether to step over the curb (decision making stage), shift their weight to one side (programming stage), and then lift the other leg to step over the curb (response output). In assessing movement performance, time and error are typically recorded. Time (speed) and error (accuracy) are frequently the criteria most indicative of performance success.

Reaction time (RT) is a measurement of the time required for a person to perceive, process, and initiate a movement in response to a stimulus. Movement time (MT) is the time between the beginning of movement in response to a stimulus signal and the completion of the response. Although RT entails no movement component, it entails the time prior to the initiation of a movement in response to a stimulus. The combination of RT and MT is referred to as response time (i.e., RT + MT = response time).

On the basis of the described theory, we designed a system that combines VR technology with motion capture devices to evaluate and train a participant’s balance control.

### Related work

In our previous work [15], we designed a three-dimensional posture frame (PF) and a human avatar in a VE. The participants who wore motion capture devices interactively manipulated an avatar to follow the fixed-shaped PF. As the PF approached the avatar in the scene, the participants had to raise one leg to conform to the shape of the PF. The width and traveling time of the PF were set as different types. The width denoted the distance between the PF boundary and the avatar. The traveling time denoted the time of the PF that approached the avatar following its appearance. Our preliminary results showed that the width and running times of the PF design affected the participants’ RT. The RT of two participants was approximately 2 seconds with a wide PF and 5 seconds of traveling time.

We then designed two PFs: static and dynamic [16]. In contrast to the static PF, the leg-raising angle and time can be manipulated in a dynamic PF. The participants were asked to lift one leg for 1 or 2 seconds at 45°or 90°. We also used a force plate to detect the center of pressure (COP) change to evaluate balance control. Four performance metrics were evaluated: mean distance-anterior posterior (MDIST-AP), mean distance-medial lateral (MDIST-ML), sway area (AREASW), and total excursions (TOTEX). Twenty-three healthy young individuals were investigated. The results indicated that for the static PF, only MDIST-AP was affected by the frame travel time (p = 0.000), whereas the other metrics were not related to the offset of the frame or the frame travel time. For the dynamic PF, an impact of the leg-raising time on MDIST-ML (p = 0.003) and TOTEX (p = 0.000) was observed.

In addition to the leg-lifting angles and times, we varied the perspectives in the present study. We hypothesized that the participant would demonstrate improved movement performance when an avatar was present in 3PP. Similar to traditional stepping exercises, small steps (smaller leg-lifting angles) and longer leg-lifting times should make leg lifting easier. Thus, we also hypothesized that the participant would exhibit improved movement performance at a smaller lifting angle and for a longer lifting time. The present study explored the influence of different perspectives on the movement performance in an interactive-visual VE.

## Methods

### Architecture

**Fig 1:**
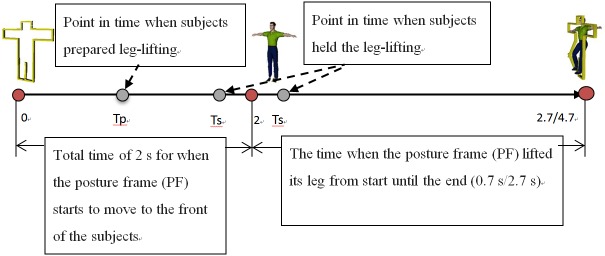
Experimental virtual environment and the parameter description of measured values. T_p_: The time when the patients prepared for leg-lifting; T_s_: The time when the patients actually started leg-lifting.

A motion capture system (Animazoo-Gypsy Gyro 18, Synertial, UK) was used to capture the participants’ movements. The system comprised 18 inertial rotational gyroscope sensors placed on the limbs and sampled them at 120 Hz. Data were delivered to a computer running motion analysis software (SIMI) and instant animation software (Motion Builder 7.5). A virtual immersive environment designed using 3D graphics software (CINEMA 4D) was developed, and a PF with or without a real-time avatar was then shown on a projection screen (Fig 1).

A foot force plate (AMTI Advanced Mechanical Technology, Inc., Watertown, MA, USA) under the participant’s feet detected the COP at a sampling frequency of 1000 Hz. The platform was connected to the computer to collect COP data, which included the COP displacement time.

### Procedure

The participants stood 2.5 m in front of the projection screen and saw a dynamic PF in the VE moving toward them. When the PF appeared after 2 seconds, the participants were asked to perform one-leg lifting (right side) without making contact with the PF. In the 3PP group, the participants saw a real-time avatar and directed the avatar through the PF. However, in the 1PP group, the participants saw only a PF and were asked to follow the PF independently (Fig 1). The PF was set for two lengths of time (0.7 and 2.7 seconds) and at two angles of knee flexion (30°and 90°), totaling four experimental conditions. Each participant performed one-leg-lifting movement under four experimental conditions, the order of which was determined using 4 × 4 balanced Latin squares to avoid the learning effect (S1 Table). After three practice trials, the mean values of the three test trials were recorded for analysis. A film of the experiment can be seen here: http://youtu.be/zMxvWcnZrZo.

### Outcome measure

The outcome measured was movement performance. The outcome parameters were the reaction time (ΔT_1_), accuracy of the movement (ΔT_2_), and ability to shift weight (Δd). The operational definitions are as follows (Fig 1):

T_p_: The time at which the participant prepared to lift the leg (i.e., COP displacement was detected, but there was no actual leg-lifting movement) after the PF had begun to move, representing the time at which the anticipatory postural reaction of the participant was observed.
T_s_: The time at which the participant actually started lifting his or her leg.
ΔT_1_: = T_s_−T_p_, which represents the participant’s reaction time.
ΔT_2_: = |T_s_ –2|, which represents the error between the point when the participant actually started leg lifting (T_s_) and the point when the PF had appeared (2 seconds into the experiment). Whether the original value is negative or positive, the absolute value represents the accuracy of the movement. The closer the absolute value is to zero, the smaller the error is. However, a negative original value (T_s_ –2) indicates that the participant started to lift his or her leg before the appearance of the PF, meaning that the participant used anticipatory postural adjustments (APAs) to complete the movement. By contrast, a positive value indicates that the participant started to lift the leg after the appearance of the PF, meaning that the participant used compensatory postural adjustments (CPAs). Thus, we also considered the sign of (T_s_− 2) in addition to its magnitude.
Δd: The COP X-Y displacement from the start to finish of leg lifting represents the ability to shift weight. If the participant transferred his or her weight completely to the left leg when lifting the right leg, the value of the COP displacement would be at a maximum.

### Experimental design

A mixed factorial design (2 × 2 × 2) was applied, where the perspective was the between-participant factor, and the leg-lifting times (0.7 and 2.7 seconds) and leg-lifting angles (30°and 90°) were the within-participant factors.

### Hypothesis

The purpose of this study was to explore the influence of different parameters on the movement performance in an interactive-visual VE. We developed the following three hypotheses (Table 1):

H1: The perspective factor (3PP vs. 1PP) influences posture performance in terms of RT (ΔT_1_), accuracy of the movement (ΔT_2_), and ability to shift weight (Δd). We expect the 3PP group to have shorter ΔT_1_, shorter ΔT_2_, and larger Δd than those of the 1PP group.
H2: The leg-lifting time factor influences posture performance in terms of RT (ΔT_1_), accuracy of the movement (ΔT_2_), and ability to shift weight (Δd). We expect a longer lifting time of the PF to cause the participants to have shorter ΔT_1_, shorter ΔT_2_, and larger Δd.
H3: The leg-lifting angle factor influences posture performance in terms of RT (ΔT_1_), accuracy of the movement (ΔT_2_), and ability to shift weight (Δd). We expect a smaller lifting angle of the PF to cause the participants to have shorter ΔT_1_, shorter ΔT_2_, and larger Δd.

**Table 1:** Hypothesis.

	DV		
IV	ΔT_1_	ΔT_2_	Δd
**perspective**	1PP>3PP	1PP>3PP	1PP<3PP
**leg lifting time (seconds)**	0.7>1>2>2.7	0.7>1>2>2.7	0.7<1<2<2.7
**leg lifting angle (°)**	30<60<90	30<60<90	30>60>90

IV: independent variables; DV: dependent variables; ΔT_1_: reaction time; ΔT_2:_ accuracy of the movement; Δd: CoP X-Y displacement of leg-lifting; 1PP: first-person perspective; 3PP: third-person perspective.

### Participants

Twenty-eight volunteers were recruited from a university. The Berg Balance Scale was used to screen for movement disorders. Those with a history of central nervous system or lower limb muscle injury, and one volunteer unable to fully participate for other reasons, were excluded. A total of 24 healthy participants (10 female and 14 male) were enrolled, with a mean age of 23.8 years (range 22–25 years). The participants were randomly divided into two groups (3PP group vs. 1PP group). The study was approved by the Institutional Review Board of National Taiwan University Hospital, and written informed consent was received from all the participants. A sample size estimate of 11 participants per group (22 total) was calculated using G-power 3.1 (Franz Faul, University of Kiel, Germany) to provide 0.84 power to detect a medium effect size of 0.4 [17], and through a repeated measures ANOVA, within-between interaction with alpha = 0.05.

### Statistical Analysis

Data were calculated in statistical means and standard deviations from three trials for each variable and each participant. First, one-way ANOVA on each of the four experimental conditions was performed to determine whether the learning effect was avoided. Second, three-way ANOVA (two within and one between factor) with repeated measures on leg-lifting times and angles was performed to assess the interaction of the perspective, leg-lifting time, and angle, and their main effects on RT, accuracy of the movement, and ability to shift weight [18]. All analyses were performed using SPSS 15.0 from PASW (SPSS, Inc., 2009, Chicago, IL, USA, http://www.spss.com/), and the significance level was set at *p* < 0.05.

## Results

**Fig 2:**
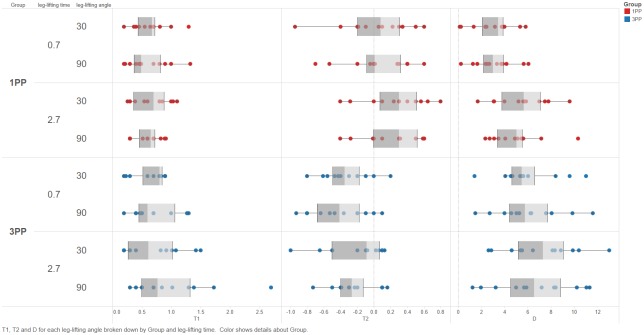
Distributions of the reaction time, the accuracy of the movement, and CoP X-Y displacement of leg-lifting for both groups in different leg-lifting angles and speed. ΔT_1_: Reaction time; ΔT_2:_ Accuracy of the movement; Δd: CoP X-Y displacement of leg-lifting. Blue dot: 3PP group; red plot: 1PP group.

In the one-way ANOVA, the order did not cause any significant effects (S2 Table), indicating that the counterbalancing was successful. Fig 2 presents the distribution of each value for the reaction time (ΔT_1_), accuracy of the movement (ΔT_2_), and COP X-Y displacement for leg lifting (Δd) for both groups (1PP vs. 3PP). In the three-way ANOVA, no significant two- or three-way interactions were noted (Table 2). Table 2 shows the reaction time (ΔT_1_) and accuracy of the movement (ΔT_2_); there were no significant main effects of the avatar, time, or angle.

**Table 2:** Three-way ANOVA cross table of reaction time (ΔT_1_), the accuracy of the movement (ΔT_2_), and CoP X-Y displacement of leg-lifting (Δd).

Source	SS	df	MS	F	*p*	ES	power
**ΔT**_**1**_							
**Between group**							
Group (1PP vs. 3PP)	0.442	1	0.442	1.446	0.242	0.062	0.210
**Within group**							
Time	0.145	1	0.145	0.848	0.367	0.037	0.143
Time*Group	0.115	1	0.115	0.671	0.421	0.030	0.123
Angle	0.097	1	0.097	0.877	0.359	0.038	0.146
Angle*Group	0.309	1	0.309	2.782	0.110	0.112	0.358
Time*Angle	0.0.078	1	0.078	1.272	0.271	0.055	0.190
Time*Angle*Group	0.086	1	0.086	1.399	0.250	0.060	0.205
**ΔT**_**2**_							
**Between group**							
Group (1PP vs. 3PP)	0.002	1	0.002	0.029	0.866	0.001	0.053
**Within group**							
Time	0.016	1	0.016	0.176	0.679	0.008	0.069
Time*Group	0.124	1	0.124	1.345	0.259	0.058	0.199
Angle	3.189E-7	1	3.189E-7	0.000	0.998	0.000	0.050
Angle*Group	0.044	1	0.044	1.211	0.283	0.052	0.183
Time*Angle	1.550E-6	1	1.550E-6	0.000	0.995	0.007	0.066
Time*Angle*Group	0.004	1	0.004	0.107	0.747	0.005	0.061
**Δd**							
**Between group**							
Group (1PP vs. 3PP)	126.304	1	126.304	6.429	0.019*	0.226	0.679
**Within group**							
Time	55.762	1	55.762	13.978	0.001*	0.389	0.946
Time*Group	10.017	1	10.017	2.511	0.127	0.102	0.329
Angle	1.116	1	1.116	0.712	0.408	0.712	0.127
Angle*Group	0.004	1	0.004	0.003	0.960	0.003	0.050
Time*Angle	2.352	1	2.352	2.215	0.151	0.091	0.296
Time*Angle*Group	0.001	1	0.001	0.001	0.980	0.000	0.050

SS: Sum of squares; df: degrees of freedom; MS: Mean Square; F: F-value; *p*: p-value; ES: effect size; power: power of the F test

1PP: first-person perspective; 3PP: third-person perspective

*p < 0.05

**Fig 3:**
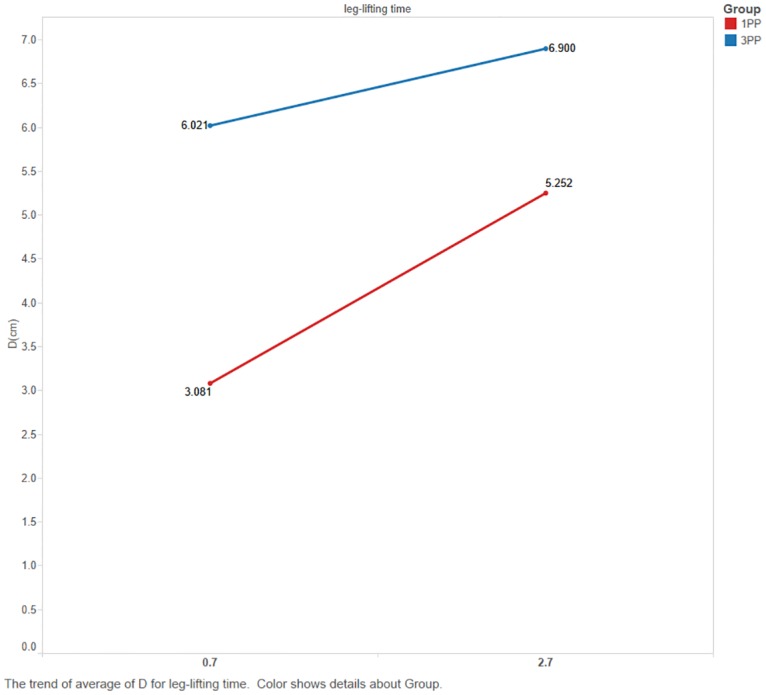
CoP X-Y displacement of leg-lifting (Δd) in different leg-lifting times for both groups. Blue line: 3PP group; red line: 1PP group.

For the ability to shift weight (Δd), the perspective had a significant main effect, F(1,22) = 6.429, *p* = 0.019. The 3PP group had a larger average displacement than that of the 1PP group. There was also a significant main effect of time, F(1,22) = 13.978, *p* = 0.001. In the 1PP group, a longer leg-lifting time corresponded to a larger COP X-Y displacement (Fig 3). The displacement was 70.5% larger for the 2.7-second time compared with the 0.7-second time. All values were lower than those of the 3PP group.

## Discussion

The main goal of the current study was to explore the influence of the perspective on the movement performance in an interactive-visual VE. We also sought to determine which parameters of the PF could influence postural control. Our results show that the perspective in VR training and the leg-lifting time of the PF can affect the ability to shift weight during a one-leg-lifting task, but not the participants’ RT or the accuracy of their movement. Our results can be applied in clinical settings, such as balance or ambulation training.

Regarding this perspective, Salamin et al. [19] reported similar results. They investigated the different effects of training participants in a ball-catching task between 3PP and 1PP perspectives. The study analyzed response times and error rates during the training. In 3PP training, the trend in error rates was similar to that in the normal daily life perspective, but not in 1PP training. For the response times, however, no significant differences were found between the perspectives. Regarding sports science, Covaci et al. [20] compared the motor performance between the two perspectives in a basketball-throwing task. Their results showed that the ball speed after 3PP training was more similar to that after real-life training than 1PP training. Overall, the findings of these studies and our own suggest that the 3PP is more effective than the 1PP in VR training for transferring motor skills to real practice. For developing the ability to shift weight for a patient recovering from stroke, a VR training program in the 3PP appears to be more effective for rehabilitation than one in the 1PP.

The results for the RT and accuracy of the movement were contrary to our hypothesis. Many factors affected the RT [21]: stimulus types, intensity, anticipation, and distractions, among many others. However, neither perspective nor the leg-lifting time or angle was associated with the RT (ΔT_1_) in our study. Some factors that we did not control for, such as psychological factors, may have influenced our results. For instance, children with motor impairment typically depend excessively on visual feedback in controlling their movement [22, 23], which may extend their reaction time. Thus, the RT may not have shown an association with different perspectives because of the healthy sample in our study.

Moreover, no associations were observed between our independent variables and the accuracy of the movement (ΔT_2_). However, we examined the data (Fig 2) and identified a compelling trend: the value is negative in 3PP, whereas it is positive in 1PP. A negative mean value indicates that the participants started to lift their leg before the 2-second mark when the PF began to move; by contrast, a positive mean value indicates that the participants started to lift their leg after the 2-second mark. Thus, in 3PP, the participants started to perform the leg-lifting movement slightly before the motion of the PF, whereas in 1PP, the participants did so immediately after the motion of the PF. On the basis of the results, we infer that the participants used APAs in 3PP but used CPAs in 1PP. Therefore, the perspective may affect the accuracy of a movement’s timing. Further research is necessary to clarify this point.

Another parameter that we manipulated is the leg-lifting time. The participants demonstrated greater ability to shift weight with a longer leg-lifting time, which was consistent with our hypothesis. Furthermore, Fig 3 shows that the participants exhibited greater ability to shift weight in 3PP. To achieve training goals, the initial training program can incorporate longer leg-lifting times. In other words, the treatment program for each patient must be **specific** and can be **adjusted** according to the **progress** of the medical condition.

Galna et al. [24] developed a Kinect-based game to enhance balance control for people with Parkinson disease. The game implemented multidirectional reaching and stepping tasks with 12 levels of difficulty, and an avatar was shown to the user. Most participants reported that they felt safe and enjoyed the games, but that some tasks were difficult. Thus, to practically apply active video games in clinical rehabilitation, designing the virtual environment in a **personalized** manner must be considered. In our study, the design of the posture frame and an avatar in 3PP can feasibly be personalized, but some parameters may have to be corrected.

As previously mentioned, we created a VE for application in clinical rehabilitation. Guo et al. [25] investigated people with or without mobility impairment and found that they respond differently to VE. In addition to objective gait and physiological response, they also investigated subjective presence. Thus, future research is required for different groups, such as older adults or patients with stroke; both subjective and objective measurement must also be considered.

## Conclusion

We hypothesized that, in an interactive-visual VE, the presence of an avatar as a visual aid and different parameters (i.e., times and angles) of one-leg lifting would influence the postural control of the participants. The participants demonstrated greater ability to shift weight when an avatar was present in the VE. The experimental results can serve as a reference for future designs of interactive VE for rehabilitation.

## Supporting Information

S1 Table Sequence of four experimental conditions in 4×4 Balanced Latin squares. n: numbers of subjects, A: 0.7second × 30°; B: 0.7 second × 90°; C: 2.7second × 30°; D: 2.7 second × 90°.
(DOCX)

S2 Table Repeated measure ANOVA table of four orders for reaction time (ΔT_1_), accuracy of the movement (ΔT_2_), and COP X-Y displacement for leg lifting (Δd). SS: Sum of squares; df: degrees of freedom; MS: Mean Square; F: F-value; p: p-value; ES: effect size; power: the power of the F test. 1PP: first-person perspective; 3PP: third-person perspective.
(DOCX)
