# Supplementary material for: Influence of the Perspectives on the Movement of One-Leg Lifting in an Interactive-Visual Virtual Environment: A Pilot Study
Source: PLoS One. 2016 Sep 20;11(9):e0163247. doi: 10.1371/journal.pone.0163247 (PMC5029811; doi:10.1371/journal.pone.0163247)
Supplement: S1 Table — n: numbers of subjects, A: 0.7second × 30°; B: 0.7 second × 90°; C: 2.7second × 30°; D: 2.7 second × 90°. (DOCX) [file pone.0163247.s001.docx]

**S1 Table. Sequence of four experimental conditions in 4×4 Balanced Latin squares**

| **Group**  (1=3pp, 0=1pp) | **n** | **Order** | **Sequence** | | | |
| --- | --- | --- | --- | --- | --- | --- |
| 1 | 3 | 1 | A | B | D | C |
| 1 | 3 | 2 | B | C | A | D |
| 1 | 3 | 3 | C | D | B | A |
| 1 | 3 | 4 | D | A | C | B |
| 0 | 3 | 1 | A | B | D | C |
| 0 | 3 | 2 | B | C | A | D |
| 0 | 3 | 3 | C | D | B | A |
| 0 | 3 | 4 | D | A | C | B |

n: numbers of subjects, A: 0.7second × 30^o^; B: 0.7 second × 90^o^; C: 2.7second × 30^o^; D: 2.7 second × 90^o^
