# Supplementary material for: Influence of the Perspectives on the Movement of One-Leg Lifting in an Interactive-Visual Virtual Environment: A Pilot Study
Source: PLoS One. 2016 Sep 20;11(9):e0163247. doi: 10.1371/journal.pone.0163247 (PMC5029811; doi:10.1371/journal.pone.0163247)
Supplement: S2 Table — SS: Sum of squares; df: degrees of freedom; MS: Mean Square; F: F-value; p: p-value; ES: effect size; power: the power of the F test. 1PP: first-person perspective; 3PP: third-person perspective. (DOCX) [file pone.0163247.s002.docx]

**S2 Table. Repeated measure ANOVA table of four orders for reaction time (△T_1_), accuracy of the movement (△T_2_), and COP X-Y displacement for leg lifting (△d)**

| Order of each within-subject variable | **SS** | **df** | **MS** | **F** | ***p*** | **ES** | **power** |
| --- | --- | --- | --- | --- | --- | --- | --- |
| **Group=3PP(n=12)** |  |  |  |  |  |  |  |
| **△T_1_** | 0.578 | 3 | 0.193 | 0.320 | 0.811 | 0.107 | 0.089 |
| **△T_2_** | 0.059 | 3 | 0.020 | 0.204 | 0.891 | 0.701 | 0.074 |
| **△d** | 88.327 | 3 | 29.442 | 1.016 | 0.435 | 0.276 | 0.187 |
| **Group=1PP(n=12)** |  |  |  |  |  |  |  |
| **△T_1_** | 0.413 | 3 | 0.138 | 1.182 | 0.376 | 0.307 | 0.213 |
| **△T_2_** | 0.287 | 3 | 0.096 | 1.053 | 0.421 | 0.283 | 0.193 |
| **△d** | 22.065 | 3 | 7.355 | 0.654 | 0.602 | 0.197 | 0.134 |
| **Not Grouped(n=24)** |  |  |  |  |  |  |  |
| **△T_1_** | 0.657 | 3 | 0.219 | 0.672 | 0.579 | 0.092 | 0.166 |
| **△T_2_** | 0.201 | 3 | 0.067 | 0.813 | 0.502 | 0.109 | 0.194 |
| **△d** | 48.699 | 3 | 16.233 | 0.637 | 0.600 | 0.087 | 0.159 |

SS: Sum of squares; df: degrees of freedom; MS: Mean Square; F: F-value; *p*: p-value; ES: effect size; power: the power of the F test

1PP: first-person perspective; 3PP: third-person perspective
